# Black-Tongue

**Published:** 1844-11

**Authors:** John F. Henry

**Affiliations:** Bloomington, Ill.


BLACK-TONGUE.—ERYSIPELAS.

BLOOMINGTON, Ill., May 20, 1844.

*Dr. W. H. Tegarden:*

DEAR SIR:—Your letter of the 16th ult., requesting my views on the nature and treatment of the disease called the '*Black Tongue*,' has remained unanswered, in consequence of my pressing engagements, growing out of the protracted prevalence of our epidemic. Having, on this day, only four cases on hand, three of which are convalescent, I propose entering into the subject with as much brevity as is consistent with a full understanding of its character and mode of treatment. And as *names* are of much importance in the practice of medicine, influencing, as you are well aware, nine-tenths of the profession, I am compelled to tell you that our disease is not the '*Black Tongue*' at all! This may somewhat abate your anxiety to know about it; but when you learn its history you will say with me that it is one of the strangest epidemics that has ever visited any portion of our country. It commenced its ravages in Pekin, on the south-east side of the Illinois river, early in the last autumn, where its mortality is said to have been very great; but it did not present itself in this vicinity until January, and then in sporadic cases of widely different character. As the season advanced the cases became numerous, and what is exceedingly singular, it seemed to fix on particular neighborhoods, where it lingered, until all who were susceptible of its influence, suffered attacks of greater or less malignity. As it declined in its first locality, other portions of the country fell under its sway. Its progress has been remarkably slow, and the space over which it has spread is very limited. It is understood that none of the towns on the north-west side of the Illinois have suffered at all, and that the extent of its ravages on the south-east side, has not exceeded a radius of 50 miles. Many believed it contagious; but this opinion has become even here, an 'obsolete idea.' The persons most liable to its attacks have been females either in the puerperal state, or advanced in years, and men of feeble or deranged health. Children and robust persons have escaped almost entirely.

The symptoms were very diverse. It almost invariably commenced with a chill succeeded by active febrile excitement, and accompanied with a variety of local affections, some of which were very troublesome, and all of which created in the minds of the people great apprehension and dread. The most common of these local affections, was an inflamed condition of the tonsils, and of the fauces generally, producing in some instances so great a swelling of the parts as to impede respiration, and almost to obstruct deglutition. This, in two instances which I have seen, extended to the tongue, enlarged it to two or three times its natural size, and this state of things has, I presume, given origin to the name, by which you have heard it called. Erysipelas of the face, commencing on the nose, and extending from this, as a centre, until the whole face and ears, and anterior portions of the head and neck are involved, was also frequent. In those cases which fell under my observation, the erysipelas appeared on the hand and fore arm. In several cases, after having appeared on the face, or in the throat, it fell on the arm pit, producing very painful tumors of the axillary glands, which, after a protracted period, terminated in suppuration. I never saw it on the body, in the first instance, except in a very mild case. In two instances it caused a single blister on the fore finger, about the size of a quarter of a dollar, so nearly resembling a burn, that my first impression was, that it had been caused by contact with the stove. In many cases, the local disease was a most painful affection of the collar bone, or the ancle bone, or of the fingers and toes without any swelling of these parts. In one case as an original disease, and in two of relapse, in one of which the disease had first assumed the character of erysipelas of the face, and in the other of sore throat, a most painful and obstinate rheumatic affection displayed itself. In one most singular case the tonsils were greatly enlarged and at the same time a very firm, but not painful tumor, of a very indolent character, extended from the ear to the clavicle, evidently composed of enlarged lymphatic glands. These were the primary local affections, but as secondary symptoms, we had inflammation of the membranes of the brain, lungs, and abdomen, the former producing stupor, the latter constituting pleurisy and peritoneal inflammation; and in several cases, suppuration of the tonsils, and of the glands of the arm-pit, and in one a deep-seated abscess beneath the fascia of the thigh.

A disease so eccentric cannot, perhaps, be designated by any name, which would be unexceptionable. That it was epidemic admits of no dispute, as in conformity to the laws which govern this class of diseases, it alone reigned during its prevalence. We know next to nothing of the causes which give a peculiar character to epidemics, but it is very certain that they are greatly under the influence of the common exciting and predisposing causes of disease. Thus women in a peculiar condition, and men of feeble health, were predisposed to our epidemic, and a period of damp, cold weather never failed to excite it in those susceptible of its influence; whereas a few days of sunny and cheerful weather put an immediate stop to its progress; and all who were sick became convalescent. I was in the habit of calling our disease *Epidemic Erysipelas*, and when the local affection was on the surface, or even in the throat or tongue, I conceived the name strictly appropriate. When some deep-seated part bore the burden of disease, the term was equally proper, though its fitness was not quite so apparent. The history of a single case will illustrate this view of the subject.

I was called to a patient with sore throat and high fever; under proper treatment the disease was dislodged from this part, and then suddenly, after a few days' respite, attacked the pleura costalis; from which it retreated to the cardiac orifice of the stomach or to the diaphragm. After hanging on this part, producing a most annoying hiccough for five days, it changed its location and appeared in genuine *Erysipelas* on the face. After spreading for two or three days, during which there was an appearance of convalescence, it disappeared and seized on the membranes of the brain, producing, as I believe is always the case when erysipelas attacks those parts, great stupor, and under the combined influence of such frequent assaults the poor fellow sank. As in all the instances of epidemic erysipelas of which we have any record, women, in the puerperal state, were most exposed to its ravages; so it has been with us: the disease in such cases uniformly appearing in the form of *puerperal fever.* For some time scarcely any escaped, unless the medical treatment was commenced before the birth of the child, and continued until the milk secretion was fully formed.

The disease was inflammatory, and demanded, throughout its early stage, an active antiphlogistic treatment. In many cases the lancet could not be dispensed with, and I was often compelled to use it repeatedly. The blood was generally buffed. Local depletion was also of inestimable value. I was in the habit of scarifying the tonsils freely, and in the two cases of swollen tongue, I plunged my lancet into it boldly. The relief afforded by the abstraction of even a small quantity of blood in this way was very remarkable. The next remedy in point of time, if not of value, was an active emetic of tartar and ipecac., and this I have repeated several times in the progress of the disease. The next in value was the free exhibition of mercurial cathartics; these were given to change the secretions of the liver, which were always, more or less, deranged. Cooke's pills or a dose of calomel, or calomel combined with Dover's powder, or opium alone, succeeded by a dose of oil or senna, after an interval of twelve hours, were the most common forms of exhibition. These were repeated and varied according to rules now well understood, until the secretions of the liver were changed. Simultaneously I directed stimulating liniments, sinapisms, or blisters to the neighborhood of the local affection, or when it appeared on the surface, the blisters were applied to the part itself. I used the mercurial ointment and lunar caustic as a local application in a few cases, but with no satisfactory results. The lancet, the emetics, the mercurial cathartics, the occasional combination of calomel and opium, and the counter-irritants were the great remedies, under a judicious use of which all cases would, I think, yield if commenced in time. The pulse ranged from 90 to 120, and in some puerperal cases to 160, and I believe in this, as in all other cases, the utility of bleeding was determined by the impression made on the pulse; when it did good it reduced its *frequency* as well as its *force.* I scarcely ever bled without making my patient sit up, an invaluable rule of practice, for which the profession is indebted to Dr. Marshall

Hall, and then I bled to incipient syncope. The tolerance of the loss of blood was in some cases great, in others but little, and I was governed in my use of the lancet by this circumstance. I should perhaps say more about puerperal cases, but there was nothing peculiar in the symptoms or treatment; the disease was, in the two cases which I saw, formed before the birth; most generally, however, it came on within three days after it, and then with a sudden chill, followed with rapid pulse, tenderness of the abdomen, headache and indomitable thirst. The lancet should be used in the first paroxysm, if at all—and hence the necessity of apprizing the friends of the danger that they may send for their medical attendant instantly on the occurrence of the chill. Calomel alone, or combined with anodyne sudorifics, could not be dispensed with, and the remark made by Dr. Gooch was confirmed by my observation, that where ptyalism took place recovery was certain. But whether this was the cause or the indication merely of amendment, I confess my inability to decide. I ought perhaps to add, that the tendency in this epidemic to abortion was very strong, and in all the cases of which I have heard any particulars, the danger from hæmorrhage was alarming. The tongue was generally heavily coated with a moist white or yellow fur, becoming in bad cases dark, dry, and cracked. All of the symptoms pointed out the great derangement of the chylopoiœtic viscera, and any practice which overlooks the morbid condition of these organs must be exceedingly defective. In a few words, the circulation should be reduced, when it is too active, by the lancet; the stomach should be emptied by emetics, and the bowels freely and repeatedly discharged by mercurial cathartics; the local affection, whatever it is, should be attended to, by its appropriate remedies, already referred to, but it so invariably followed the condition of the system, that an improvement of this never failed to remove them.

I should not omit to state, that I saw a number of cases in which, during the progress of the disease, after the subsidence or removal of the more active stages, the circulation became so languid that I was compelled to resort to stimulants and tonics in free doses. But this took place most generally in cases which had been suffered to run their course pretty much without control. I would add further, that when the face was the seat of erysipelas the danger was generally much less, than when it siezed on the throat, and in either situation the danger was comparatively less than when it fixed on a vital part. The danger in all cases was clearly indicated by the pulse; when this remained but slightly affected the disease was generally mild. I may add, that I saw no tendency in wounds or accidents to produce erysipelatous inflammation. While the disease was upon our borders, and during its prevalence, I saw a case of compound fracture of the leg, and one of gun-shot wound, besides having performed three operations of an important character, to say nothing of having used the lancet many times a day, and in none did I witness the slightest tendency to the disease. The only exception was an old lady who had been badly burned, and who had heretofore suffered with erysipelas.

In conclusion, I ought to state, that when the disease assumed the rheumatic form, I found no change of remedies required, except a greater reliance on blisters, and a more free use of sudorific anodynes, combined with mercurials.

Should you deem this communication of any use to the people of Kentucky, to whom I am under so many obligations for past kindness, I cannot object to its publication, although it has been hastily and somewhat carelessly written.

I am, respectfully, yours, &c.

JOHN F. HENRY.

